# Intravitreal injections: past trends and future projections within a UK tertiary hospital

**DOI:** 10.1038/s41433-021-01646-3

**Published:** 2021-06-25

**Authors:** Reena Chopra, Gabriella C. Preston, Tiarnan D. L. Keenan, Pádraig Mulholland, Praveen J. Patel, Konstantinos Balaskas, Robin D. Hamilton, Pearse A. Keane

**Affiliations:** 1 NIHR Biomedical Research Centre for Ophthalmology, Moorfields Eye Hospital NHS Foundation Trust and UCL Institute of Ophthalmology, London, UK; 2 Division of Epidemiology and Clinical Applications,, National Eye Institute, National Institutes of Health, Bethesda, MD UK; 3 Centre for Optometry and Vision Science Research, School of Biomedical Sciences, Ulster University, Coleraine, UK

**Keywords:** Drug therapy, Retinal diseases

## Abstract

**Aims:**

To describe past trends and future projections for the number of intravitreal injections being administered at a large tertiary hospital in London, United Kingdom.

**Methods:**

Retrospective data from Moorfields Eye Hospital were collected using the electronic medical record system. Descriptive statistics were used to visualise overall trends. Time series forecasting was used to predict the number of injections that will be administered up to and including the year 2029.

**Results:**

The number of injections has increased nearly 11-fold from 2009 to 2019, with a total of 44,924 injections delivered in 2019. The majority of injections were given for the treatment of neovascular age-related macular degeneration. Aflibercept formed 87% of injections administered in 2019. The number of injections is predicted to continue to increase every year, with nearly 83,000 injections forecasted in the year 2029.

**Conclusion:**

The demand for intravitreal injections has increased substantially over the last decade and is predicted to further increase. Healthcare systems will need to adapt to accommodate the high demand. Other solutions may include longer-acting therapies to reduce the treatment burden.

## Introduction

The availability of intravitreal therapy for the treatment of retinal disease has expanded substantially over the last decade and is the first-line treatment for neovascular macular disease [1] and foveal-involving macular oedema. Anti-vascular endothelial growth factor (anti-VEGF) drugs form the majority of intravitreal therapeutics and are administered as frequently as every four weeks, often for many years.

Several anti-VEGF drugs have undergone regulatory approvals. Pegaptanib was the first anti-VEGF drug to be licensed by the Food and Drug Association (FDA) for the treatment of neovascular AMD in the USA in 2004. It was subsequently approved by the European Medicines Agency (EMA) in 2006 [2]. However, pegaptanib was not recommended as a treatment for neovascular AMD by the National Institute for Health and Care Excellence (NICE) [3]. Ranibizumab (Lucentis, Genentech, San Francisco, California, USA/Novartis Pharmaceuticals, Basel, Switzerland), shown to be more effective than pegaptanib, was the second anti-VEGF drug to be licensed by the FDA for the treatment of neovascular AMD in the USA in 2006 [4], by the EMA in 2007 [2], and became the first anti-VEGF drug to obtain NICE approval in August 2008 [3]. Aflibercept (Eylea, Regeneron, Tarrytown, New York, USA, and Bayer, Berlin, Germany) was subsequently the third anti-VEGF drug to receive licensing by the FDA in 2011 [5], and by the EMA in 2012 [6], and was approved by NICE in July 2013 [7]. Bevacizumab (Avastin, Genentech/Roche), also an anti-VEGF drug, has shown to be as efficacious as ranibizumab [8, 9] but has not been licensed for intravitreal use. It is however often used in many other parts of the world due to its significantly lower cost price or is used for indications falling outside of current licensing regulations. In China, the anti-VEGF drug conbercept was approved for neovascular AMD in 2013 after promising preclinical studies, with phase III studies still in progress. Anti-VEGF drugs were first approved for neovascular AMD, but have since been widely approved for several other common conditions such as choroidal neovascularisation secondary to myopia, and macular oedema secondary to diabetes or vein occlusion. Other available intravitreal therapeutics include steroids, anti-virals, recombinant protease enzymes, and anti-tumour necrosis factor drugs.

At Moorfields Eye Hospital (MEH), a large tertiary referral centre in the United Kingdom, the Retinal Therapy Unit (RTU) was established in 2008 as an outpatient clinic to deliver these injections. In February 2016, Moorfields introduced a larger RTU to accommodate the increasing number of patients that would benefit from treatment. The demand for treatment is still growing and is associated with several factors. Most treated conditions, particularly neovascular AMD, are highly age-dependent. With an ageing population, the number of individuals at risk of these conditions and requiring treatment is increasing substantially. In addition, as new drugs continue to be developed, and receive approval for a growing number of conditions, the trajectory of the number of patients requiring treatment is expected to increase even further, particularly if drugs are approved for more common conditions such as dry AMD. Furthermore, within each condition, there is an increasing spectrum of indications. For example, neovascular AMD is being treated at an earlier stage in patients with better visual acuity than in the past, and recent trials have shown proliferative diabetic retinopathy may be successfully delayed with intravitreal therapy [10]. Thus, it is crucial to predict the demand for these therapies to ensure healthcare systems can accommodate patients who require sight-saving treatment without delay.

We report the number of intravitreal injections being delivered at MEH, including one large centre (City Road) and several smaller satellite sites. We forecast the number of injections that will be delivered over the next 10 years given the current trend. We also report on the conditions presenting to the RTU and how this has evolved over time.

## Methods

### Dataset

Weekly and monthly injection data were extracted from the MEH electronic medical record system in an anonymised format. Data were aggregated from one large teaching hospital (City Road), and several smaller eye units operated by Moorfields (Croydon University Hospital, Darent Valley Hospital, Ealing Hospital, Loxford Polyclinic, Northwick Park Hospital, Potters Bar Community Hospital, St. Ann’s Hospital, St. George’s Hospital, Sir Ludwig Guttmann Health and Wellbeing Centre). All sites follow the same treatment protocols. Data included the number of patients receiving unilateral or bilateral injections, the conditions being treated, and the number of drugs being administered, per week and month at each site.

Conditions were grouped by age-related macular degeneration (AMD), diabetic macular oedema (DMO), retinal vein occlusion (RVO), myopia, and others (or unknown). The other category also contains patients that were given treatment for AMD, DMO, or RVO but were not within the NICE criteria for treatment [1, 11–15]. Injection drugs included ranibizumab, bevacizumab, aflibercept, and others (intravitreal steroid [e.g. triamcinolone], intravitreal steroid implant [e.g. dexamethasone, fluocinolone acetonide], ocriplasmin, foscarnet, adalimumab).

This study was conducted in accordance with the Declaration of Helsinki and the UK’s Data Protection Act. Permission for data collection and analysis was provided through registration as a clinical audit (CA17/MR/28). The only retrospective anonymised data were used without the active involvement of patients.

### Statistical analysis

Descriptive statistics were used to summarise the data. The dataset was stratified by drug and condition. For the latter, we analysed how many visits comprised bilateral injections. Data analysis was performed in Python 3.6. Forecasting analysis was performed to predict the number of monthly injections that will be delivered up to the end of the year 2029. This was carried out using the open-source Prophet package [16, 17], an additive regression model, which uses historical injection data to fit future trends. For this model, historical monthly data between 2008 and 2019 was input to the model. The output was a forecast of the number of injections per month between January 2020 and December 2029, inclusive, with 95% confidence intervals. The model automatically removes outliers and considers seasonality effects, for example, the model accounts for the reduced service that operates every December due to the holiday season. For the purpose of this analysis, only the number of injections delivered per month were fed into the model, and excluded other data such as patient demographics.

## Results

### Total number of injections

The first recorded injection was given in August 2008, with only a total of 361 injections given in that year. Between August 2008 and December 2019 inclusive, 18,100 patients and 22,211 unique eyes received a total of 252,263 injections. In 2009, the first full year of operation, 4143 injections were delivered to 1375 patients (1513 eyes, 8717 patient attendances). The total number of injections increased almost 11-fold to 44,924 injections (8617 patients, 10,258 eyes, 46,520 patient attendances) in 2019.

**Fig. 1:**
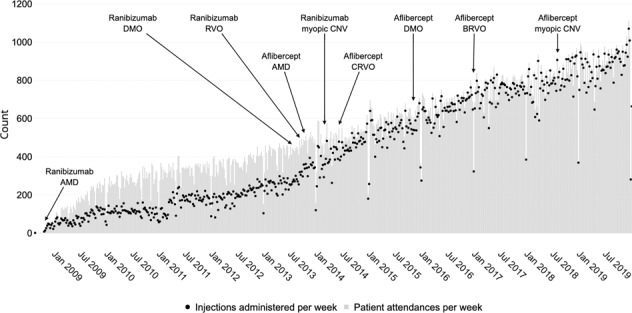
Historical trends. Number of patients that attended the intravitreal injection clinic every week (black), and the number of injections administered (grey), between August 2008 to December 2019 (inclusive), at Moorfields Eye Hospital. The annotations highlight when drugs became available on the Moorfields formulary and for which conditions.

Figure 1 shows the number of patient attendances and the number of injections delivered per week. The greatest number of injections administered in a single week was 1071 in the second week of December 2019––one of the last few data points collected. Between 2008 and 2012, while ranibizumab was the only NICE-approved drug for the treatment of neovascular AMD [3] and was provided on a pro re nata treatment regime, the average ratio of patients to injections was 1.95:1. Aflibercept was introduced in 2013 [7], licensed for up to 12-weekly doses, and commonly provided using fixed dosing in the first year of treatment followed by a treat and extend regimen [18]. Consequently, patients were switched onto this drug and the ratio of patients to injections reduced to 1.14:1, indicating that the majority of patients received treatment at every visit. Figure 1 clearly shows the shift between 2013 and 2014 from pro re nata treatment, with a low ratio of injections to patient attendances, to treat and extend with a high ratio of injections to patient attendances. The number of injections given per week per site is shown in Supplementary Figures 1 and 2.

The monthly number of patients entering the RTU and exiting (i.e. last appointment in the RTU) is shown in Supplementary Figure 3. The ratio is also presented (number exiting divided by the number entering). A ratio of <1 means that more patients are entering than exiting. While the ratio has been increasing over time, it remained <1 until August 2019, meaning more patients had their last appointments in the RTU in the last third of 2019.

### Conditions receiving therapy

**Fig. 2:**
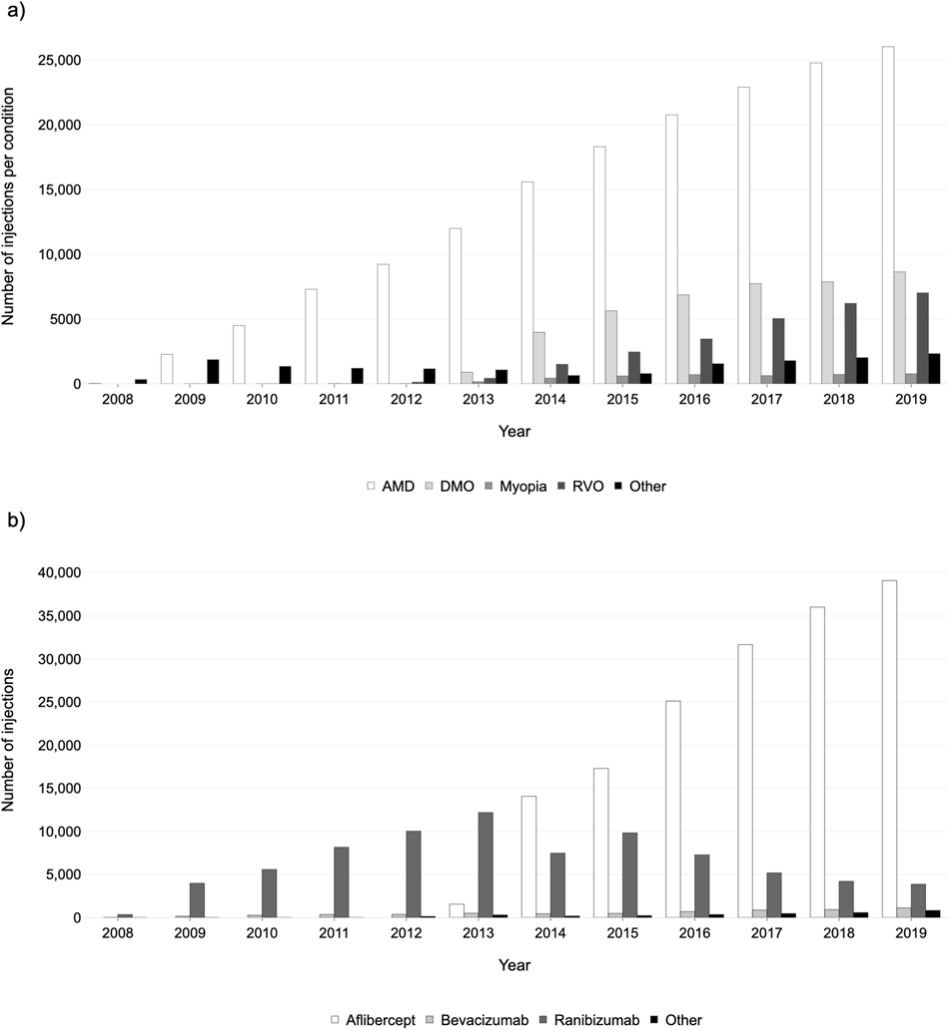
Number of injections given per year from 2008–2019. **a** Stratified by retinal condition, (**b**) stratified by drug.

Neovascular AMD was by far the most commonly treated condition, accounting for 67.4% of all injections in 2019, and as high as 87.7% in 2012 (Fig. 2a). The number of injections provided for neovascular AMD has increased every year, but with a declining growth factor. Between 2012 and 2013, a relative increase of 30.1% was observed, whereas only a relative change of +5.0% was seen between 2018 and 2019. In 2019, 26,048 injections were delivered for neovascular AMD.

**Fig. 3:**
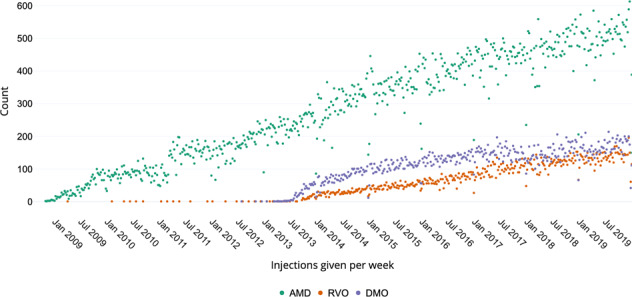
Weekly number of injections per condition. Number of injections given per week for neovascular age-related macular degeneration (AMD), macular oedema secondary to retinal vein occlusion (RVO), and diabetic macular oedema.

Anti-VEGF drugs were approved for use in DMO and RVO in early 2013 [11, 13], and their use in these conditions has risen steadily. The proportion of all injections that were prescribed for DMO was 17.2% in 2014 and rose slightly to 19.2% in 2019. Whereas, RVO accounted for 6.8% of injections in 2014, and more than doubled to 15.9% in 2019. Ranibizumab was approved for the treatment of myopic choroidal neovascularisation (CNV) in late 2013 [19] and was available on the Moorfields formulary in early 2014. In 2014, only 2.0% of injections were given for this myopic CNV. In 2019, the proportion reduced slightly to 1.7%. The weekly number of injections given for neovascular AMD, RVO, and DMO are presented in Fig. 3. The percentage of treated retinal conditions per site is provided in Supplementary Table 1.

In 2009, 1894 injections were given for ‘other’ conditions. A significant proportion of this is likely to be attributable to neovascular AMD, where treatment eligibility was limited and conditioned on the availability of funding. The number of injections within this category reduced every year until 2014. From 2015, the number of injections falling into this category followed an upward trajectory, with 2324 injections being performed in 2019, which may be attributable to conditions such as idiopathic CNV, radiation retinopathy, neovascular glaucoma, pre-vitrectomy in eyes with proliferative diabetic retinopathy, and more.

### Intravitreal drugs

Ranibizumab accounted for over 95% of all injection drugs from 2008 to 2012 inclusive (Fig. 2b). The use of aflibercept grew rapidly after it received market authorisation for neovascular AMD [7] and was available on the Moorfields formulary in 2013. Patients with neovascular AMD were switched from ranibizumab to aflibercept in 2013 and 2014. However, ranibizumab was the only drug licensed for the treatment of macular oedema and myopic CNV and thus an increase in ranibizumab use was seen in 2015. A similar switch from ranibizumab to aflibercept was seen for other conditions as aflibercept was approved for more indications. Aflibercept was available on the Moorfields formulary for central RVO in mid-2014, DMO in late-2015, branch RVO in early-2017, and myopic CNV in mid-2018. In 2019, aflibercept was administered in 87.0% (*n* = 39,096) of injections, whereas only 8.6% (*n* = 3866) of injections were ranibizumab. The use of bevacizumab and other drugs continues to remain low. The distribution of drugs administered per site is provided in Supplementary Table 2.

### Bilateral injections

**Fig. 4:**
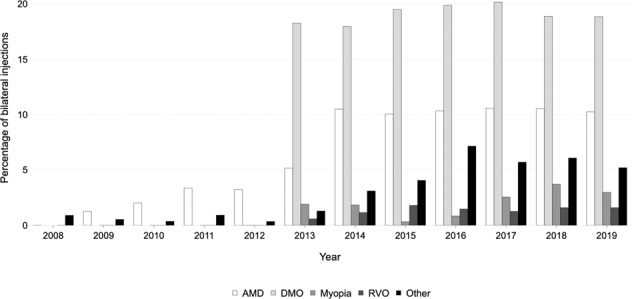
Bilateral treatment. Percentage of patient visits where injections were given bilaterally, stratified by retinal condition and year.

Between 2012 and 2019, bilateral injections were performed at an average of 19.2% visits for those receiving treatment for DMO (Fig. 4). From 2009 to 2013, bilateral injections were only performed at 3.0% of visits for patients with neovascular AMD. This rose sharply between 2014 and 2019, bilateral injections were given at an average of 10.4% of visits. This proportion appears to have plateaued for both DMO and AMD despite the increasing number of injections being given for these conditions. Bilateral injections were only given to a smaller percentage of patients who had myopic CNV or RVO. Other conditions that received bilateral injections included retinal dystrophies and uveitis.

### Forecasting the number of injections

**Fig. 5:**
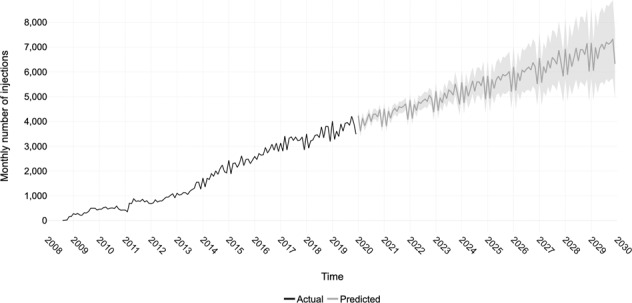
Forecasting future trends. Actual number of monthly injections from 2008 to the end of 2019 (black). Forecasted number of monthly injections for a 10-year period from 2020 to the end of 2029 with shaded 95% confidence intervals (grey).

The forecasting analysis estimated that, at the current trend, the number of injections will incrementally rise every year but with a declining growth factor (Fig. 5, Table 1). At the current rate, the number of injections that will be delivered in 2029 is ≈82,876 (95% confidence intervals: 65,540–100,214).

**Table 1:** Actual number of injections delivered per year at Moorfields Eye Hospital from years 2008 to 2019.

Year	Actual number of injections	Forecasted number of injections	95% confidence intervals		Year-over-year growth factor
			Lower bound	Upper bound	
2008	361				
2009	4143				11.48
2010	5850				1.41
2011	8518				1.46
2012	10,529				1.24
2013	14,565				1.38
2014	22,178				1.52
2015	27,842				1.26
2016	33,434				1.20
2017	38,193				1.14
2018	41,726				1.09
2019	44,924				1.08
2020		49,192	46,639	51,742	1.10
2021		52,778	49,976	55,504	1.07
2022		56,537	53,022	60,008	1.07
2023		60,296	55,592	65,024	1.07
2024		64,291	57,941	70,784	1.07
2025		67,823	59,741	76,148	1.05
2026		71,582	61,404	81,976	1.06
2027		75,341	62,894	87,840	1.05
2028		79,390	64,564	94,278	1.05
2029		82,867	65,540	100,214	1.04

Forecasted number of injections from 2020 to 2029. Growth factor is calculated using actual and forecasted number of injections, using the formula number of injections in the year divided by the number of injections in the previous year.

## Discussion

In this report, we summarise the trends in the delivery of intravitreal injections at a large tertiary ophthalmic hospital in London, United Kingdom. The injection service has grown in capacity substantially over the last 10 years. Our forecast analysis predicts that if the current trend continues over the next 10 years, nearly 83,000 injections will be administered in the year 2029.

The most common indication for intravitreal injections at Moorfields Eye Hospital is for the treatment of neovascular AMD, and the second most common indication being DMO, though this varies by site. For example, at Ealing Hospital, injections are as commonly indicated for DMO as they are for AMD. This is likely explained by the variation in the demographics of the populations at each site such as age, ethnicity, and social determinants of health, which are known to be risk factors for retinal diseases [20]. Both of these conditions are increasing in prevalence and are likely to lead to an increasing need for injections. In Europe, the incidence of late AMD is expected to increase to 700,000 per year by 2050 due to population growth and lengthening life expectancy [21]. Similarly, the prevalence of diabetes is increasing with the changes in lifestyle and diets. In England alone, the total number of adults with diabetes is projected to rise to 4.6 million in 2030––an increase of 9.5% from 2010 [22]. A significant proportion of these will benefit from intravitreal therapy. Minassian et al. estimated that 7.1% of individuals with diabetes in England had DMO in one or both eyes––of these, 38.9% were estimated to have clinically significant DMO [23]. Similarly, Keenan et al. found 13.9% of patients managed for diabetic eye disease in the hospital had clinically significant DMO [24]. Thus, it is essential for hospital systems to prepare for the increasing demand to ensure injections can be provided promptly as delay in treatment is known to affect visual prognosis in AMD and DMO [25, 26]. Longer-acting agents may be one solution to the predicted demand. Newer anti-VEGF agents such as brolucizumab may soon be widely available for patients which may require less frequent therapy [27]. Port-delivery systems that can be implanted and refilled with anti-VEGF, and thus aim to reduce treatment burden, are in clinical trials and have shown positive results [28, 29]. These systems may allow for up to 6 months between treatments and thus reducing the frequency of visits to the eye clinic.

The surge in the number of injections being delivered over the last 10 years is likely multifactorial. Although likely to play a role, the number of injections cannot solely be explained by an increasing prevalence of disease as the rate of uptake of injections has exceeded the prevalence rates. As expected, the number of injections increased more sharply at junctures where drugs were approved for DMO and RVO. In addition, the hospital expanded significantly in February 2016 to cope with and in preparation for the increasing demand. The creation of this extra capacity and the ability to treat more retinal conditions led to an increased uptake of injections at a higher rate than in prior years. Although the number of injections being administered is still increasing year on year, the growth factor appears to be declining. This may indicate that the hospital is reaching its ceiling of available capacity, the prevalence of disease could be stabilising. While the number of new patients entering the RTU is still greater than the number exiting the clinic, the gap between the two is reducing. It has also been postulated that the age-specific incidence of AMD is decreasing [30]. Future increases in the growth factor might be a consequence of the significant increase in disease prevalence or licensing of drugs for use in further conditions. For example, clinical trials are currently underway investigating the effectiveness of intravitreal pegcetoplan [31] and avacincaptad pegol [32] for geographic atrophy. As geographic atrophy has a higher prevalence than neovascular AMD, it is likely that a large population could potentially benefit from these drugs if approved.

Our forecasting analysis was derived using only historical data without controlling for other variables such as the estimated future prevalence of disease. With an ageing population in the UK, the prevalence of age-dependent diseases such as AMD are likely to increase. In London alone, the population is expected to increase by 8.5% from 8.9 million individuals in 2019 to 9.66 million in 2029 [33]. Over a third of the London population in 2019 will be over 50 years old––an increase of 500,000 individuals from 2019. While the forecasting did not account for this factor specifically, the model accounts for historical trends of which population is one factor. The described future population trend has also been observed in the last decade and therefore will be accounted for in historical trends. In addition, in light of the COVID-19 crisis and the impact of isolation measures, the number of injections performed at Moorfields in April 2020 fell significantly [34]. In addition, the last third of 2019 showed that more patients were exiting the RTU than new patients entering, which may be explained by the reduction in activity due to the pandemic. Although the forecast for 2020 will be inaccurate in absolute numbers, it may still accurately reflect the number of eyes that would have required treatment. The actual future trajectory will likely be influenced by multiple factors such as changes in treatment regimens and protocols, the availability of novel therapies that may be longer-acting, and the provision to treat more retinal conditions. In addition, both an increased awareness of macular disease by the public, and the introduction of optical coherence tomography into primary eye care [35], may improve the detection rate of conditions requiring referral and subsequent treatment into the hospital eye service.

The emergence and upward trajectory in the uptake of intravitreal therapies for the treatment of multiple retinal conditions illustrate the dramatic speed of adoption, particularly in conjunction with NICE approvals. In prior analysis of trends of intravitreal injections in England, Keenan et al. similarly observed a substantial increase in intravitreal injections for AMD following NICE approval for ranibizumab [36]. At Oslo University Hospital, Norway, a similar trend was seen in the uptake of injections from 2006 to 2019, with neovascular AMD forming a similar percentage of injections [37]. While the mainstay of treatment at this institution is bevacizumab, there was a significant uptake in aflibercept as a second-line treatment for neovascular AMD and first-line treatment for DMO. Our figures clearly illustrate the increase in aflibercept at Moorfields since it received NICE approval and was available on the formulary in 2013. As the licensing permits 12-weekly administration using a treat and extend regimen, the majority of patients were switched to aflibercept from ranibizumab, with the latter being recommended on a pro re nata regimen and overall requiring more frequent visits compared to treat and extend posology [38]. Visual acuity treatment outcomes have been shown to be similar between both ranibizumab and aflibercept in neovascular AMD [39]; however, the need for fewer injections and clinic visits is beneficial for a system where the demand is high and growing. Aflibercept is currently licensed for several conditions including neovascular AMD and macular oedema secondary to diabetes or vein occlusion. Both anti-VEGF treatments require long-term administration with many patients remaining within these clinics indefinitely, particularly for the treatment of neovascular AMD [40].

In conclusion, these findings show the dramatic speed of adoption as intravitreal drugs are approved and become available for additional retinal conditions. The potential introduction of intravitreal therapy for other more prevalent conditions such as geographic atrophy would place further significant demand. As the upward trend is expected to continue, resources will need to be adequately allocated towards service provision. Here, innovation in new therapies, including longer-acting therapies, and automation may be necessitated to mitigate the increasing demand in the assessment and treatment of patients. In addition, forecasting future trends may aid healthcare systems to plan resources and secure funding to be able to accommodate the expected demand.

### Summary

#### What was known before

- Intravitreal drugs are the first-line of treatment for many retinal diseases. Their use has expanded over the last decade.

#### What this study adds

- Intravitreal injections were adopted with dramatic speed over the last decade. As demand is forecasted to increase further, healthcare systems will need to increase their capacity, or administer longer-acting agents that require fewer clinic visits.

## Supplementary information

Supplementary Material
