# Supplementary material for: Intravitreal injections: past trends and future projections within a UK tertiary hospital
Source: Eye (Lond). 2021 Jun 25;36(7):1373–8. doi: 10.1038/s41433-021-01646-3 (PMC8227364; doi:10.1038/s41433-021-01646-3)
Supplement: Supplementary file 1 — Supplementary Material [file 41433_2021_1646_MOESM1_ESM.docx]

# Supplementary Material

#


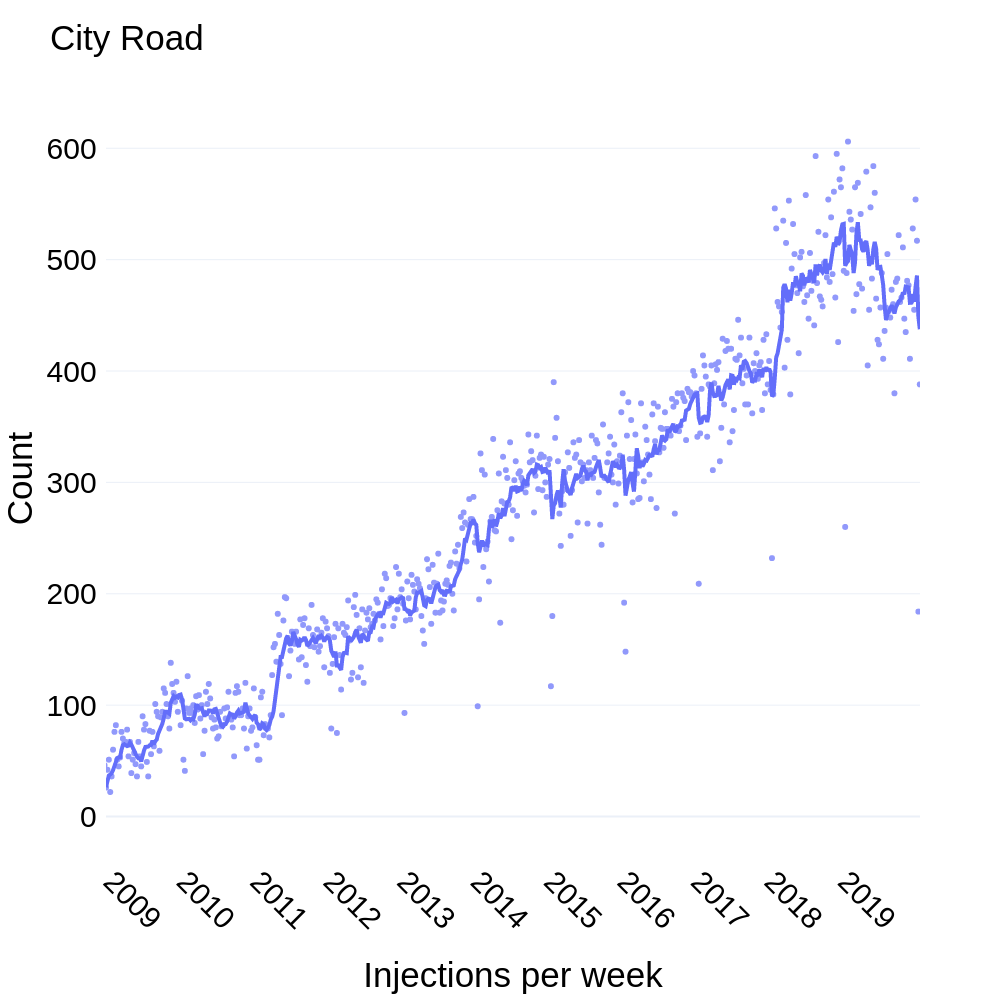


**Supplementary Figure 1.** Number of injections delivered per week at City Road, the main site of Moorfields Eye Hospital. Moving average line is shown, calculated using an 8-week window length.


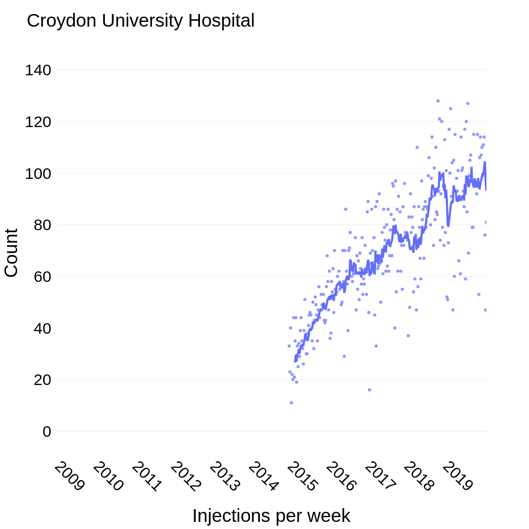

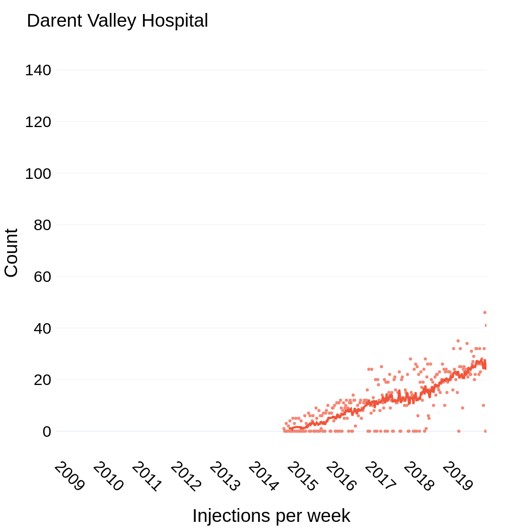

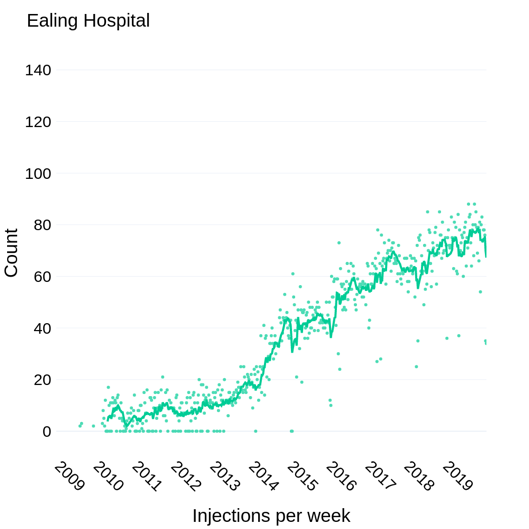

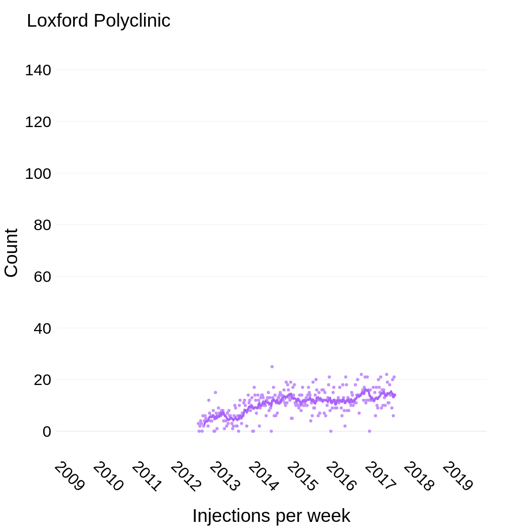

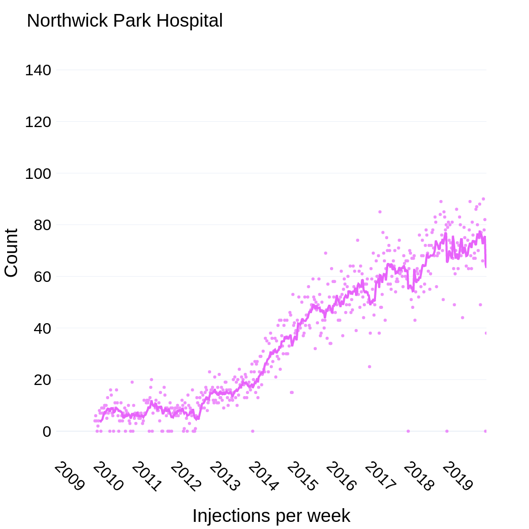

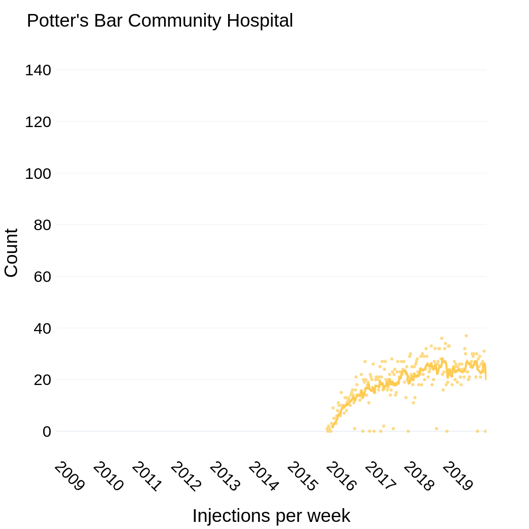

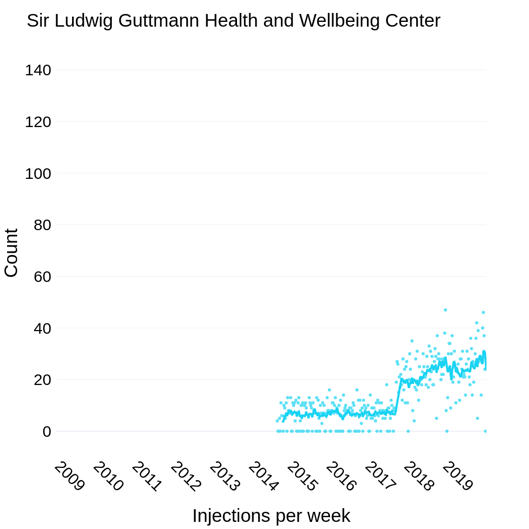

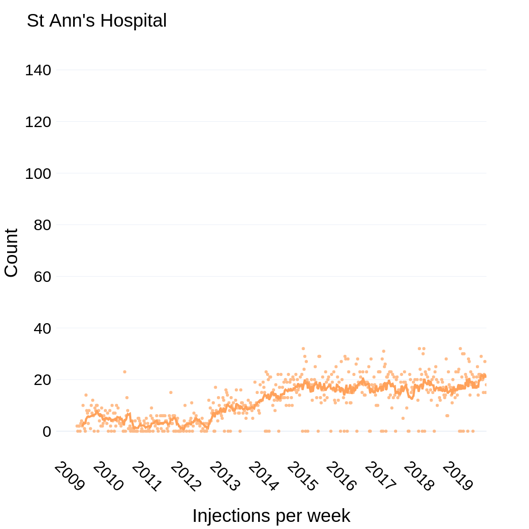

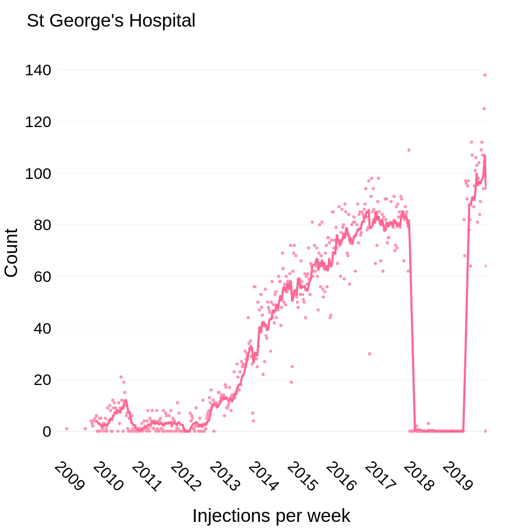


**Supplementary Figure 2.** Number of injections given per week at Moorfields Eye Hospital satellite sites. Moving average lines are shown, calculated using an 8-week window length.


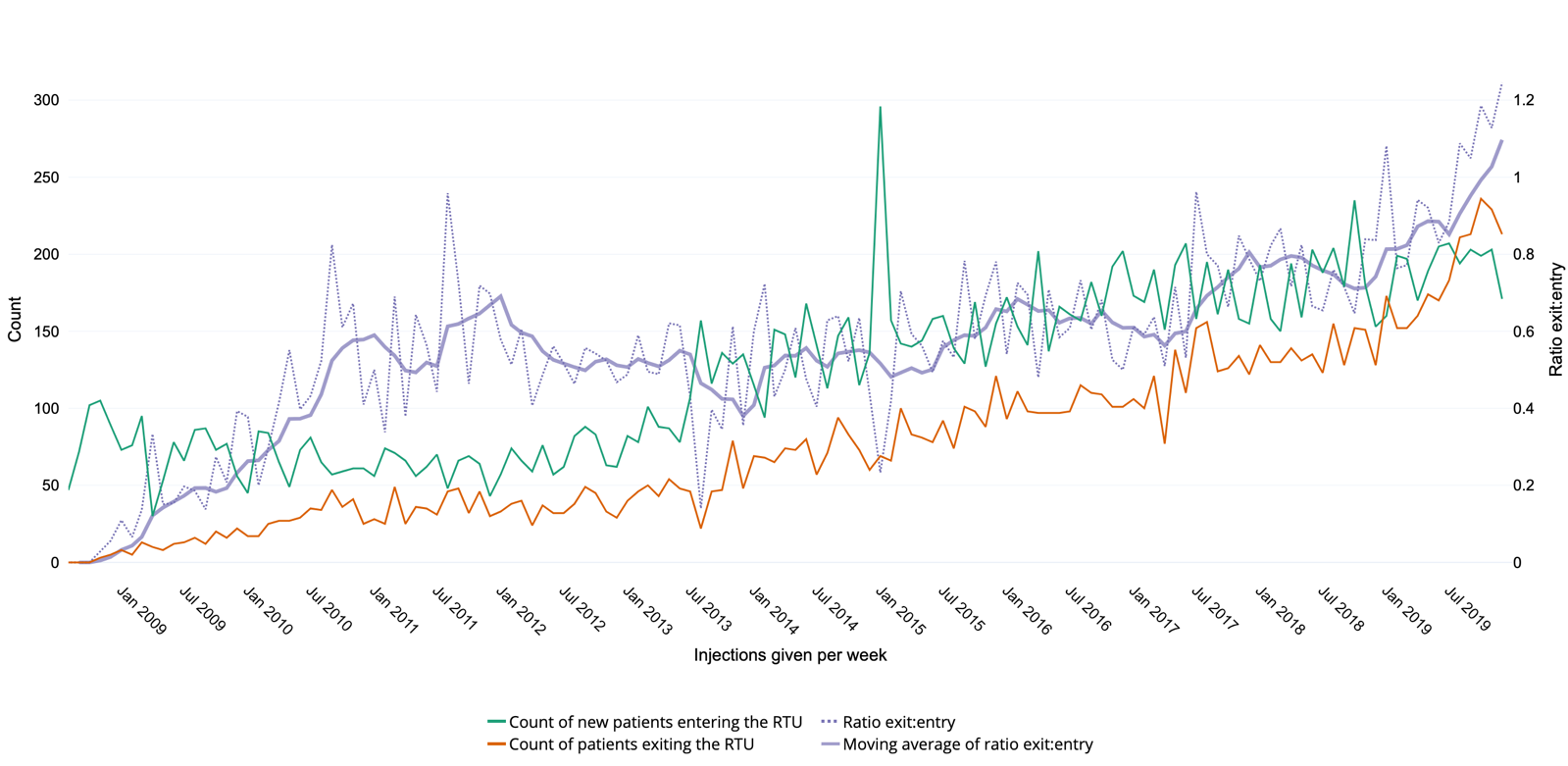


**Supplementary Figure 3.** Count of patients entering and exiting the RTU. The purple dotted line shows the ratio between exit and entry. A ratio >1 means that more patients are exiting the clinic than new patients entering. The moving average is calculated using a 6-month window length.

|  | **Percentage of AMD / RVO / DMO / other** | | | | | | | | | |
| --- | --- | --- | --- | --- | --- | --- | --- | --- | --- | --- |
| Year | City Road | Croydon University Hospital | Darent Valley Hospital | Ealing Hospital | Loxford Polyclinic | Northwick Park Hospital | Potters Bar Community Hospital | Sir Ludwig Guttmann Health and Wellbeing Center | St Ann's Hospital | St George's Hospital |
| 2008 | 9.1 / 0.0 /  0.0 / 90.9 |  |  |  |  |  |  |  |  |  |
| 2009 | 53.5 / 0.0 /  0.0 / 46.4 |  |  | 100.0 / 0.0 /  0.0 / 0.0 |  | 100.0 / 0.0 /  0.0 / 0.0 |  |  | 81.0 / 0.0 /  0.0 / 19.0 | 100.0 / 0.0 /  0.0 / 0.0 |
| 2010 | 75.7 / 0.1 /  0.0 / 24.1 |  |  | 100.0 / 0.0 /  0.0 / 0.0 |  | 100.0 / 0.0 /  0.0 / 0.0 |  |  | 82.9 / 0.0 /  0.0 / 17.1 | 100.0 / 0.0 /  0.0 / 0.0 |
| 2011 | 85.8 / 0.1 /  0.0 / 14.1 |  |  | 100.0 / 0.0 /  0.0 / 0.0 |  | 100.0 / 0.0 /  0.0 / 0.0 |  |  | 90.7 / 0.0 /  0.0 / 9.3 | 100.0 / 0.0 /  0.0 / 0.0 |
| 2012 | 87.9 / 1.2 /  0.0 / 10.8 |  |  | 86.1 / 0.9 /  0.0 / 13.0 | 93.5 / 0.0 /  0.0 / 6.5 | 82.7 / 0.8 /  0.0 / 16.5 |  |  | 92.4 / 0.0 /  0.0 / 7.6 | 86.8 / 3.7 /  0.0 / 9.5 |
| 2013 | 83.7 / 3.2 /  4.0 / 9.0 |  |  | 66.4 / 2.7 /  25.0 / 5.9 | 89.3 / 3.6 /  0.9 / 6.2 | 86.1 / 1.0 /  2.3 / 10.6 |  |  | 93.2 / 0.6 /  0.0 / 6.2 | 68.7 / 2.8 /  25.0 / 3.5 |
| 2014 | 75.8 / 5.1 / 13.1 / 6.0 | 64.3 / 13.2 / 18.6 / 3.9 | 70.0 / 0.0 /  20.0 / 10.0 | 43.0 / 8.0 /  46.3 / 2.8 | 76.5 / 18.0 /  2.6 / 2.9 | 74.2 / 4.0 /  20.5 / 1.3 |  | 22.4 / 62.1 / 15.5 / 0.0 | 74.6 / 11.6 / 12.8 / 1.1 | 52.9 / 11.4 / 32.4 / 3.4 |
| 2015 | 71.2 / 6.8 / 15.1 / 6.8 | 66.7 / 11.5 / 18.9 / 3.0 | 69.7 / 21.4 /  6.9 / 2.1 | 38.3 / 9.0 /  50.2 / 2.6 | 74.5 / 14.5 /  9.6 / 1.4 | 68.1 / 7.7 /  22.4 / 1.8 | 75.0 / 25.0 /  0.0 / 0.0 | 27.6 / 56.1 / 14.2 / 2.0 | 66.3 / 12.9 / 19.1 / 1.7 | 57.3 / 10.9 / 28.5 / 3.4 |
| 2016 | 67.3 / 8.6 / 14.6 / 9.5 | 57.1 / 12.1 / 21.0 / 9.9 | 69.1 / 22.0 / 6.9 / 1.9 | 37.9 / 10.4 / 49.8 / 1.9 | 73.3 / 19.0 /  7.2 / 0.5 | 61.2 / 9.6 /  27.4 / 1.7 | 77.6 / 15.7 / 6.4 / 0.3 | 44.5 / 38.5 / 12.6 / 4.3 | 58.8 / 13.9 / 25.8 / 1.5 | 58.2 / 12.2 / 26.6 / 2.9 |
| 2017 | 64.2 / 11.2 / 15.9 / 8.7 | 55.8 / 13.3 / 20.4 / 10.4 | 63.3 / 23.7 / 11.1 / 1.9 | 39.0 / 14.3 / 45.1 / 1.7 | 65.1 / 23.8 / 11.1 / 0.0 | 59.2 / 12.6 / 26.7 / 1.5 | 79.0 / 17.0 / 2.8 / 1.1 | 55.0 / 32.2 / 10.6 / 2.3 | 51.8 / 19.5 / 26.8 / 2.0 | 57.6 / 16.9 / 23.3 / 2.3 |
| 2018 | 62.4 / 14.5 / 15.1 / 8.0 | 53.0 / 14.1 / 23.9 / 9.0 | 71.5 / 17.5 / 7.4 / 3.6 | 41.0 / 14.9 / 42.2 / 1.8 |  | 58.2 / 14.1 / 25.4 / 2.2 | 76.8 / 18.0 / 1.5 / 3.7 | 56.9 / 28.4 / 13.1 / 1.6 | 49.9 / 16.0 / 31.8 / 2.3 | 59.8 / 13.7 / 12.0 / 14.5 |
| 2019 | 60.9 / 15.1 / 14.5 / 9.5 | 52.8 / 13.1 / 28.0 / 6.2 | 72.5 / 20.4 / 4.9 / 2.2 | 41.6 / 16.8 / 39.3 / 2.3 |  | 55.4 / 15.0 / 26.9 / 2.7 | 76.8 / 17.5 / 1.6 / 4.0 | 57.2 / 28.8 / 11.5 / 2.5 | 50.5 / 19.0 / 24.8 / 5.6 | 54.8 / 17.8 / 24.7 / 2.8 |

**Supplementary Table 1.** Percentage of treated retinal conditions per year and per site.

|  | **Percentage of ranibizumab / aflibercept / other** | | | | | | | | | |
| --- | --- | --- | --- | --- | --- | --- | --- | --- | --- | --- |
| Year | City Road | Croydon University Hospital | Darent Valley Hospital | Ealing Hospital | Loxford Polyclinic | Northwick Park Hospital | Potters Bar Community Hospital | Sir Ludwig Guttmann Health and Wellbeing Center | St Ann's Hospital | St George's Hospital |
| 2008 | 96.1 / 0.0 / 3.9 |  |  |  |  |  |  |  |  |  |
| 2009 | 96.2 / 0.0 / 3.8 |  |  | 100.0 / 0.0 / 0.0 |  | 69.6 / 0.0 / 30.4 |  |  | 100.0 / 0.0 / 0.0 | 100.0 / 0.0 / 0.0 |
| 2010 | 94.6 / 0.0 / 5.4 |  |  | 99.2 / 0.0 / 0.8 |  | 99.7 / 0.0 / 0.3 |  |  | 100.0 / 0.0 / 0.0 | 100.0 / 0.0 / 0.0 |
| 2011 | 95.5 / 0.0 / 4.5 |  |  | 98.0 / 0.0 / 2.0 |  | 100.0 / 0.0 / 0.0 |  |  | 100.0 / 0.0 / 0.0 | 100.0 / 0.0 / 0.0 |
| 2012 | 95.0 / 0.0 / 5.0 |  |  | 95.4 / 0.0 / 4.6 | 100.0 / 0.0 / 0.0 | 99.0 / 0.0 / 1.0 |  |  | 100.0 / 0.0 / 0.0 | 96.2 / 0.0 / 3.8 |
| 2013 | 82.9 / 11.0 / 6.1 |  |  | 88.1 / 8.2 / 3.6 | 88.3 / 11.7 / 0.0 | 89.3 / 10.7 / 0.0 |  |  | 88.5 / 11.5 / 0.0 | 89.2 / 9.0 / 1.8 |
| 2014 | 28.2 / 68.5 / 3.2 | 50.0 / 50.0 / 0.0 | 20.0 / 70.0 / 10.0 | 61.0 / 38.1 / 0.9 | 40.1 / 58.3 / 1.6 | 38.0 / 61.8 / 0.1 |  | 72.7 / 20.7 / 6.6 | 27.2 / 72.8 / 0.0 | 45.9 / 54.0 / 0.2 |
| 2015 | 30.9 / 66.1 / 3.0 | 35.0 / 64.5 / 0.5 | 27.6 / 71.0 / 1.4 | 60.4 / 38.8 / 0.8 | 32.2 / 65.8 / 2.1 | 39.8 / 59.8 / 0.5 |  | 49.7 / 43.3 / 7.0 | 31.2 / 68.8 / 0.0 | 39.3 / 60.4 / 0.3 |
| 2016 | 21.0 / 74.8 / 4.1 | 23.5 / 76.0 / 0.5 | 39.2 / 60.3 / 0.5 | 29.1 / 69.5 / 1.4 | 25.2 / 72.1 / 2.7 | 24.6 / 75.2 / 0.2 | 11.2 / 88.8 / 0.0 | 39.2 / 55.0 / 5.8 | 17.8 / 82.2 / 0.0 | 16.0 / 83.9 / 0.0 |
| 2017 | 13.2 / 82.0 / 4.8 | 17.3 / 81.7 / 1.0 | 25.2 / 73.8 / 0.9 | 10.2 / 87.8 / 2.0 | 19.4 / 79.5 / 1.0 | 14.1 / 85.7 / 0.3 | 21.9 / 73.8 / 4.4 | 20.6 / 76.6 / 2.7 | 9.1 / 90.9 / 0.0 | 10.3 / 89.3 / 0.5 |
| 2018 | 9.5 / 85.8 / 4.7 | 10.9 / 87.5 / 1.6 | 10.8 / 89.1 / 0.1 | 6.7 / 91.4 / 1.9 |  | 10.9 / 88.8 / 0.3 | 27.0 / 72.0 / 0.9 | 11.7 / 85.3 / 2.9 | 5.2 / 94.8 / 0.0 | 8.7 / 89.3 / 1.9 |
| 2019 | 9.2 / 84.7 / 6.1 | 8.4 / 90.0 / 1.6 | 4.1 / 94.9 / 1.0 | 6.8 / 90.3 / 2.9 |  | 10.0 / 89.5 / 0.5 | 17.0 / 79.5 / 3.5 | 7.9 / 88.7 / 3.3 | 4.5 / 95.5 / 0.0 | 4.8 / 94.5 / 0.7 |

**Supplementary Table 2.** Percentage of ranibizumab / aflibercept / other drugs of total number of injections administered per year per site.
